# Vehicular Network Intrusion Detection Using a Cascaded Deep Learning Approach with Multi-Variant Metaheuristic

**DOI:** 10.3390/s23218772

**Published:** 2023-10-27

**Authors:** Ankit Manderna, Sushil Kumar, Upasana Dohare, Mohammad Aljaidi, Omprakash Kaiwartya, Jaime Lloret

**Affiliations:** 1 School of Computer and Systems Sciences, Jawaharlal Nehru University, New Delhi 110067, India; ankit97_scs@jnu.ac.in (A.M.); skdohare@mail.jnu.ac.in (S.K.); 2 School of Computing Science & Engineering, Galgotias University, Greater Noida 203201, India; upasana.dohare@galgotiasuniversity.edu.in; 3 Computer Science Department, Faculty of Information Technology, Zarqa University, Zarqa 13110, Jordan; mjaidi@zu.edu.jo; 4 Department of Computer Science, Nottingham Trent University, Nottingham NG11 8NS, UK; 5 Computing and Informatics Research Centre, Nottingham Trent University, Nottingham NG11 8NS, UK; 6 Instituto de Investigación para la gestión Integrada de Zonas Costeras, Universitat Politécnica de Valencia, Camino Vera s/n, 46022 Valencia, Spain; jlloret@dcom.upv.es

**Keywords:** VANET, intrusion detection, deep learning, long short-term memory, convolution neural network

## Abstract

Vehicle malfunctions have a direct impact on both human and road safety, making vehicle network security an important and critical challenge. Vehicular ad hoc networks (VANETs) have grown to be indispensable in recent years for enabling intelligent transport systems, guaranteeing traffic safety, and averting collisions. However, because of numerous types of assaults, such as Distributed Denial of Service (DDoS) and Denial of Service (DoS), VANETs have significant difficulties. A powerful Network Intrusion Detection System (NIDS) powered by Artificial Intelligence (AI) is required to overcome these security issues. This research presents an innovative method for creating an AI-based NIDS that uses Deep Learning methods. The suggested model specifically incorporates the Self Attention-Based Bidirectional Long Short-Term Memory (SA-BiLSTM) for classification and the Cascaded Convolution Neural Network (CCNN) for learning high-level features. The Multi-variant Gradient-Based Optimization algorithm (MV-GBO) is applied to improve CCNN and SA-BiLSTM further to enhance the model’s performance. Additionally, information gained using MV-GBO-based feature extraction is employed to enhance feature learning. The effectiveness of the proposed model is evaluated on reliable datasets such as KDD-CUP99, ToN-IoT, and VeReMi, which are utilized on the MATLAB platform. The proposed model achieved 99% accuracy on all the datasets.

## 1. Introduction

The security and safety of the VANET depend on an intrusion detection system (IDS). Vehicles with On-Board Units (OBU) and Road-Side Units (RSU) can broadcast vital information, such as traffic conditions, road dangers, and safety alerts, using Mobile Ad-hoc Networks (MANETs) [1,2]. While VANET has many advantages for intelligent transportation systems, its open and dynamic nature also leaves them subject to several security risks, such as malicious attacks, breaches in data integrity, and unauthorized access [3,4]. In VANETs, an IDS’s main objective is to quickly identify and address such security risks, minimizing their potential influence on network operations and ensuring vehicle and passenger safety [5,6]. In order to be effective, an IDS must handle the particular difficulties provided by VANET, such as the high vehicle mobility, the constrained computational capacity of OBU and RSU, and the dynamic network topology [7,8]. IDS that are based on misuse and IDS that are based on anomalies are the two major types used most frequently in VANET. The former is dependent on predetermined patterns or known attack signatures. The IDS sounds an alarm when these patterns are present in network traffic. Misuse-based IDS are effective at spotting well-known attacks, but they may struggle to deal with brand-new or unidentified attack patterns [9]. Furthermore, it can be resource-intensive to maintain and update the signature database.

However, anomaly-based IDS do not rely on pre-established patterns. Instead, based on past data, they construct a model of typical network behavior [10,11]. An anomaly is indicated when observed behavior departs significantly from the model. This method is helpful in identifying new attacks, but it can also produce false positives due to valid differences in network behavior [12]. Several important factors need to be taken into account in order to improve IDSs performance in VANET. IDS must, above all, be able to detect security risks in real time in order to act quickly. Particularly in safety-critical settings, delays in attack detection and mitigation might have serious repercussions. Second, given the constrained computational capabilities of OBU and RSU, IDS should be as light and resource-efficient as possible to reduce the impact on vehicle performance [13]. Data privacy is yet another crucial factor to take into account. IDS must respect user privacy and refrain from gathering personal data in order to detect intrusions [14,15]. In order to maintain their integrity and efficiency, IDS must also be shielded from attacks. The dependability of IDS in VANET must be maintained through secure communication routes. The use of collaborative detection can improve IDSs functionality in VANET. Collaborative IDSs can more efficiently identify threats by allowing numerous cars and RSUs to share information. Deep Learning, in particular, has shown promise in improving IDS in VANET using machine learning (ML) approaches [16]. IDSs based on ML may learn from network dynamics and adapt to changing attack patterns, making them more resistant to new threats. Additionally, ML-based IDS automates feature extraction and selection, requiring less human involvement and exposing confidential data. The growing significance of VANET in enabling intelligent transport systems and improving traffic safety is the driving force behind this study. VANET has demonstrated significant promise for enhancing communication between vehicles and infrastructure, preventing collisions, and effectively managing traffic flow.

**Figure 1:**
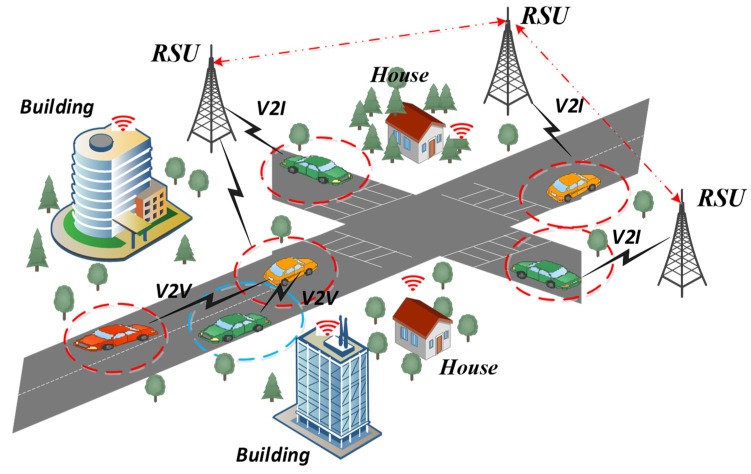
VANET Architecture.

In VANET, the major concern is the presence of various types of attacks, including DoS and DDoS. The effectiveness and safety of intelligent transportation systems may be harmed by these attacks, which could prevent the network from operating normally. Because VANETs operate in a dynamic, decentralized way and rely on wireless communication among vehicles, they are distinct and difficult environments for maintaining security. This wireless nature exposes them to potential eavesdropping, data tampering, and unauthorized access. Moreover, the rapid and unpredictable changes in network topology due to vehicle movement make it essential to establish secure and stable communication channels. Figure 1 represents the VANET Architecture. These security issues seriously threaten the dependability and security of VANET because any communication breakdown might have detrimental effects on traffic safety and the entire transportation network. There is a critical requirement for a strong and high-level security system to handle these problems and guarantee the ongoing growth of intelligent transportation systems. To address this issue, an AI-based NIDS is proposed, leveraging deep learning techniques for effectively detecting and preventing attacks. The proposed NIDS seeks to provide increased capabilities for detecting and mitigating various types of assaults in the VANET environment by integrating AI and deep learning techniques, thereby enabling intelligent transportation systems with improved traffic safety and collision prevention capabilities.

(1) To enhance the NIDS’s capability to detect sophisticated attack patterns, this paper proposes using the CCNN model. By leveraging CCNN, the NIDS effectively extracts high-level features from VANET data, enabling better identification of intricate network behaviors and potential anomalies.
(2) To achieve more precise classification results, this paper introduces SA-BiLSTM. SA-BiLSTM considers long-range dependencies in sequential data crucial for VANETs with extended temporal patterns, leading to reduced false positives and more reliable intrusion detection.
(3) To further enhance the NIDS’s efficiency, this paper utilizes MV-GBO to fine-tune the CCNN and SA-BiLSTM components. This optimization leads to better convergence during training, resulting in a more effective and accurate intrusion detection system for VANETs.

The rest of this paper is organized as follows: The literature studies conducted for VANET-NIDS are discussed in Section 2. The proposed methodology is discussed in Section 3. In Section 4, the results obtained using the projected model are discussed, and this paper is concluded in Section 5.

## 2. Related Work

For Vehicle Ad Hoc Networks (VANET) in 2020, Zhou et al. [17] suggested a distributed collaborative intrusion detection system, DCDIV, to address security vulnerabilities brought on by malicious attacks. To create stable and dependable communication linkages between vehicles, DCDIV used a reputation-based cooperative communication mechanism. Dynamic behavior analysis was then used to find malicious behaviors based on invariants that had been mined. Simulation results show that DCDIV performs better than existing techniques with greater detection rates, lower false alarm rates, and faster attack detection, maintaining system security throughout the detection process. Using a hidden generalized mixture transition distribution model (HgMTD) for VANET, Liang et al. [18] used FM-HgMTD in 2020. With the use of the multi-objective optimization (NSGA-II) algorithm and the expectation-maximization (EM) method, FM-HgMTD efficiently filtered messages from nearby cars to cut down on overhead and detection time. It also accurately predicted and detected malicious communications. GaDQN-IDS was a brand-new Bayesian game theory and Deep Q-learning NIDS for VANETs, which Liang et al. [19] introduced in 2022. In order to balance efficiency and accuracy, GaDQN-IDS modeled the interactions between the IDS and attackers as a dynamic intrusion detection game. Through Deep Q-learning Network (DQN) Adjustment and Error Priority Learning (EPL), the IDS could change the tradeoff or undergo retraining based on detection performance and driving conditions. The works discussed were performed in a simulated environment using tools like NS2, SUMO, and OpenStreetMap, and the authors were skeptical about how they would perform in a real-life environment.

Deep neural network (DNN)-based anomaly detection system for VANET was presented by Alladi et al. in 2021 [20]. The framework was made to deal with the growing number of linked vehicles as well as the many kinds of anomalies that could happen in the network. On RSU, DNN architectures were used to categorize communication sequences as aberrant or real. Using Cooperative Intelligent Transport Systems (C-ITS) over VANET, Ercan et al. [21] offered a novel Machine Learning (ML) mechanism for Intrusion Detection Systems (IDS) in 2022. To better detect position falsification attacks, a serious security risk in C-ITS, the technique made use of three new features connected to the sender position. The work employed Ensemble Learning (EL) further to enhance detection performance and compared two ML techniques for classification, k-Nearest Neighbour (kNN) and Random Forest (RF). The dataset considered for this work was a public dataset, but we did not use another dataset to validate the work. A collaborative IDS for VANET based on machine learning and privacy preservation was presented by Zhang and Zhu [22] in 2018. The suggested algorithm used the dual-variable perturbation technique with the alternating direction method of multipliers to train a classifier for intrusion detection while preserving privacy through dynamic differential privacy. The work fails to discuss the need for evolving threat resolution, as the frequent update and adapt method is the requirement for IDS in VANETs. This paper also struggles to find the balance between security and privacy. In 2021 [23], Raja et al. suggested Secure and Private-Collaborative IDS (SP-CIDS) for VANET. In order to improve the storage efficiency, accuracy, and scalability of the IDS, SP-CIDS used distributed machine learning based on the Alternating Direction Method of Multipliers (ADMM) in conjunction with vehicle-to-vehicle cooperation. The system employed Differential Privacy (DP) techniques to protect confidential information while collaborating to satisfy privacy concerns. Singh et al. [24] research on the Industrial Internet of Things (IIoT) in 2021 focused on the crucial security issue in VANET. Increasing VANET security is essential due to the possible threats to life and the smooth operation of the network. A novel approach that combines conventional IDS and honeypots is offered to meet the issues of intrusion detection. This approach aims at identifying both known and unexpected threats while optimizing resource utilization. The work demonstrated honeypot-based IDS solutions but did not validate them with a real-world dataset. In order to address the vulnerability of Inter-Vehicle Communications (IVC) in the intelligent routing of Electric Vehicles (EVs) [25] for dynamic wireless charging, Kosmanos et al. [26] offered a probabilistic cross-layer IDS based on ML approaches in 2020. In order to maintain the security and dependability of EV communication networks during intelligent routing and dynamic charging situations, the suggested IDS attempts to identify and mitigate cyber assaults, such as spoofing. An automated, secure framework for continuous cloud service availability in smart connected vehicles was introduced in 2019 by Aloqaily et al. [27]. It combined an intrusion detection system with high-quality service delivery to prevent security assaults. Trusted third-party organizations arbitrate communication between requesters and providers, and smart vehicles are grouped into groups that specialize in particular services. For intrusion detection, machine learning and data traffic analysis were used. The suggested remedy was to improve the effectiveness and security of cloud services for smart cars in smart cities. To tackle the issue of IDS, a Deep learning-based NIDS approach is proposed for the detection and prevention of attacks, using two public datasets to work on and validate.

## 3. Proposed Methodology

**Figure 2:**
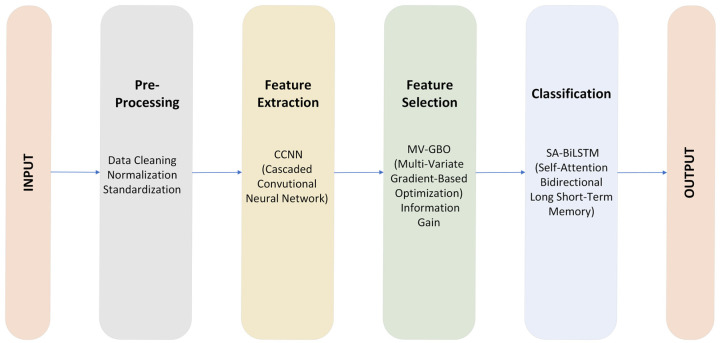
Proposed Classification Model for IDS.

This study developed an innovative AI-based NIDS for VANET. The system leverages deep learning techniques, such as CCNN for feature learning and SA-BiLSTM for classification. The aim is to effectively detect and counteract various attacks, ensuring the security and reliability of intelligent transportation systems on VANETs. Table 1 represents the abbreviations used in the following sections:

The workflow depicted in Figure 2 illustrates our data processing pipeline. It starts with the initial input data, which goes through the preprocessing phase, including data cleaning, normalization, and standardization. The next step involves feature extraction using a cascaded convolutional neural network (CCNN), feature selection using multi-variate gradient-based optimization (MV-GBO), and information gain, and then the optimized data enters the SA-BiLSTM classifier, resulting in the final execution, as shown in Algorithm 1.
**Algorithm 1:** Functioning of the overall model1.InputReceive raw data as vector input.2.Pre-processingData Cleaning: Remove noise and irrelevant information.Normalization: Scale numerical features to a standard range.Standardization: Shifting the distribution of each feature to have a mean of zero and a standard deviation of one.3.Feature Extraction using CCNN (Convolutional Complex Neural Network):Apply CCNN to extract essential features from the pre-processed data.4.Feature Selection using MV-GBO Based Information Gain:Apply Multi-Variate Gradient-Based Optimization (MV-GBO) to select the most informative features.5.Classification using SA-BiLSTM (Self-Attention Bidirectional Long Short-Term Memory):Use the selected features and apply SA-BiLSTM for classification.6.OutputGenerate the results with class labels.

### 3.1. Pre-Processing

In this study, the pre-processing stage is applied to the initial data. To improve the quality and diversity of the data, pre-processing is conducted by performing data cleaning, normalization, and standardization. The collected data are passed as input to the pre-processing phase.

#### 3.1.1. Data Cleaning

Before being analyzed by the intrusion detection system in VANETs, data cleaning is a crucial pre-processing technique used to improve the quality and reliability of the data. This procedure involves locating and fixing errors, addressing missing data, reducing noise, and ensuring data consistency. The aim is to guarantee that the data utilized for intrusion detection are of a high standard and free from errors or inaccuracies that can result in false positives or false negatives. Due to imperfect sensor data collection, transmission problems, and other considerations, data acquired in VANETs may contain errors. To increase the correctness of the dataset, data cleaning involves locating and fixing these flaws. Due to delays in connectivity or malfunctioning sensors, some data points in VANETs could be lost. Data cleaning involves managing these missing values by removing incomplete data or employing attribution, among other methods. Random changes or errors that might not be relevant to intrusion operations are called noise in the data. Data points that stand out from the rest are known as outliers. For intrusion detection, data cleaning removes noise and controls outliers so that it may concentrate on important trends. The data in VANETs may originate from several sources, resulting in differences in formats or units of measurement. Data standardization promotes data uniformity and makes the data suitable for intrusion detection analysis.

#### 3.1.2. Normalization

Data normalization is a crucial technique used in intrusion detection for VANET. It involves transforming data into a standardized format, typically by scaling it to a specific range or distribution. This process ensures consistency and comparability across different features, enhancing the performance of machine learning models and other detection methods. In the context of VANETs, where security is paramount, normalization aids in identifying patterns and anomalies that may indicate potential attacks. Normalization improves feature comparisons and convergence during model training by making the data consistent and removing biases due to varying scales. Additionally, it enhances the system’s ability to handle outliers and sets consistent detection thresholds, resulting in more effective and accurate intrusion detection in VANETs.

#### 3.1.3. Standardization

In intrusion detection for VANET, data standardization is a crucial technique. The data undergoes a transformation process to a value of zero and a standard deviation of one, thus standardizing the dataset and facilitating consistent and uniform comparisons amongst features. By standardizing the data, machine learning models can better discern patterns and anomalies associated with potential intrusions into VANET. This preprocessing step allows the models to learn from uniform data, leading to improved detection accuracy. Data standardization is beneficial in VANETs, where security is essential, as it aids in identifying outliers and abnormal behavior that may indicate malicious activities. Data standardization enhances the effectiveness of intrusion detection systems, contributing to a safer and more secure vehicular communication environment. The preprocessed data are given as input to CCNN for high-level feature learning.

### 3.2. Feature Extraction

A Deep Learning architecture called a Cascaded Convolutional Neural Network (CCNN) is made up of several CNNs [28] connected in succession. A hierarchical structure is created when the output of one CNN is used as the input for the following CNN. The numerous layers of characteristics that each CNN layer learns and extracts from the input data enable the network to recognize increasingly intricate and esoteric patterns. When learning hierarchical representations is required for the job, a cascaded CNN is required. A shared hidden layer segment of the network that extracts common traits required by succeeding subnetworks is divided into a global atmospheric light estimate subnetwork that uses the outputs of the shared hidden layer to map the global atmospheric light. Our cascaded CNN architecture allows it to predict both the global atmospheric light and the medium transmission at the same time. The common hidden layer consists of four convolutional layers with ReLU nonlinearity and a 3 × 3 × 16 filter size.

Input Layer: The input to the first CNN is denoted as X.

First CNN Layer: The first CNN layer consists of convolutional operations, followed by a non-linear activation function. It learns basic features from the input data:

(1) Output1=ReLU(w1×X+b1)

where *w*1 is the weight matrix of the convolutional filters in the first CNN layer, X is the input data, and *b*1 is the bias term of the first CNN layer.

Second CNN Layer: The output of the first CNN is passed as input to the second CNN layer, which learns more complex features:

(2) Output2=ReLU(w2×Output1+b2)

where *w*2 is the weight matrix of the convolutional filters in the second CNN layer, and *b*2 is the bias term of the second CNN layer. By stacking multiple CNN layers in a cascade, the cascaded CNN can learn hierarchical representations of the input data. After extracting the relevant features, the MV-GBO-based IG technique performs feature selection, enhancing classification performance for intrusion detection in VANETs.

### 3.3. Information Gain Computation via Entropy Analysis

The statistical distribution of feature weights in information gain is based on correlations between features and classifications. Assume that A ($a_{1},\;a_{2},\;\ldots \ldots ,\;a_{p})$ is a collection of p features, F ($f_{1},\;f_{2},\;\ldots \ldots ,\;f_{n})$ is a group of n data points, and B ($b_{1},\;b_{2},\;\ldots \ldots ,\;b_{m})$ is a group of m type labels. The proportion of classes in F with the label “$P_{i}$” is shown by the number $P({Cl}_{i})$ (where i=1, 2,…, m). Entropy is given as per Equation (3).

(3) $$H\left(B\right)=-\sum_{i=1}^{m}P({Cl}_{i})\log_{2}P({Cl}_{i})$$

Now, for each feature $I_{j}$ in the dataset (where *j* is an index running from 1 to *p*), the associated conditional entropy, considering its values $\left(I_{j}^{1},I_{j}^{2},\ldots \ldots ,I_{j}^{k}\right)$, is shown in Equation (4).

(4) $$H\left(B|I_{j}\right)=-\sum_{q=1}^{k}P\left(I_{j}^{q}\right)\sum_{i=1}^{m}P({Cl}_{i}|I_{j}^{q})\log_{2}P({Cl}_{i}|I_{j}^{q})$$

Here, $I_{j}^{q}$ represents the $q^{th}$ distinct value the $j^{th}$ feature can assume, and *k* is the total number of such distinct values. $P(I_{j}^{q})$ signifies the prior probability of category variable Cl for that value. The conditional probability of variable Cl after considering the attribute $I_{j}$ is given by $P\left({Cl}_{i}|I_{j}^{q}\right)$. Since the difference between $H\left(B\right)$, $H\left(B|I_{j}\right)$, which is determined by Equation (4), is what determines the value of the information obtained from the attribute $I_{j}$, it can be inferred from the formula shown in Equation (5).

(5) $$IG(I_{j})=H(B)-H(B|I_{j})$$

#### 3.3.1. MV-GBO

A brand-new metaheuristic optimization tool called the GBO algorithm combines population-based and gradient-based approaches. It efficiently explores the full search space using a collection of vectors and two operators. GBO seeks to discover the best answers for a given set of search metrics by imitating population-based, gradient-based, and Newtonian approaches.

##### Initialization Phase

During optimization, the GBO algorithm uses the control parameters (α) and probability rate (β) to balance between exploitation and exploration. The number of iterations and the size of the population are adapted according to the complexity of the problem being solved. In GBO, the solution space is represented by a vector of N vectors in D-dimensional space. These initial vectors are generated randomly within the D-dimensional search space, as per Equation (6).

(6) $$x_{n}=x_{min}+r(0,\;1)\times (x_{max}-x_{min})\;$$

where r (0, 1) denotes a random number within the range [0, 1], and $x_{min}$ and $x_{max}$ denote the decision variable x lower and upper bounds, respectively.

##### Gradient Search Rule (GSR)

The GBO method uses a key component to achieve a balanced exploration of important search space regions while getting close to global and near-optimal spots. The use of ρ is described in terms of Equations (7)–(9).

(7) ρ1=2×r×α−α 

(8) $$\alpha =\left|\beta \times sin(3\pi /2)+sin(\beta \times 3\pi /2)\;\right|$$

(9) $$\beta =\beta_{min}+(\beta_{max}-\beta_{min})\times {\left((1-{c/T)}^{3}\right)}^{2}\;$$

where, $\beta_{min}$ and $\beta_{max}$ are constant values of 0.2 and 1.2, respectively. T is the total number of iterations, while the variable c stands for the current iteration’s number. Based on the sine function, the parameter ρ1 is in charge of balancing exploration and exploitation. It fluctuates dynamically throughout the optimization process, beginning with a high value to promote a wide range of solutions and progressively falling over iterations to hasten convergence. The method effectively investigates a wide range of alternative solutions by increasing the parameter value through specified iterations within a range. This approach enhances the GBO algorithm’s ability to efficiently search and find optimal solutions while maintaining a balance between exploitation and exploration. GSR (Global Search Radius) can be calculated using Equation (10).

(10) $$gsr=r\times \rho 1\times 2∆x\times x_{n}\;/\;(x_{worst}-x_{best}+\varepsilon )\;$$

Random behavior is employed to create a randomized exploration mechanism, facilitating the discovery of local optima. The variable ∆x changes with iterations, as shown in Equations (11) and (13). A random number (r) is introduced to enable exploration.

(11) ∆x=r(1 : N)×|step| 

(12) $$step=(x_{best}-x_{r1}^{c})\;/\;2+\delta \;/\;2\;$$

(13) $$\delta =2\times r\times \left|x_{r1}^{c}+x_{r2}^{c}+x_{r3}^{c}+x_{r4}^{c}\;/\;4-x_{n}^{c}\right|$$

where, r(1 : N) represents a random vector in the range [0, 1]. The GSR calculation incorporates these random factors to support a well-rounded exploration process, allowing the GBO algorithm to effectively explore potential solutions, including local optima, in the search space. Four independent integers (r1, r2, r3, and r4) are randomly chosen in the GBO algorithm so that (r1≠r2≠ r3≠ r4≠ n). The difference between $x_{best}$ and $x_{r1}^{c}$ serves as a measure of the phase scale that the step represents, as shown in Equation (12). Directional movement is used to drive the vectors ($x_{n}$) towards convergence throughout the solution field in order to obtain convergence. dm is introduced to move $x_{n}$ in direction of the best vector $\left(x_{best}-x_{n}\right)$ and computed as per Equation (14).

(14) $$dm=r\times \rho 2\times (x_{best}-x_{n})\;$$

where, ρ2 is a random parameter that is utilized to modify the phase size of each vector agent. r is a uniformly distributed value in the range [0, 1]. ρ2 is calculated as per Equation (15).

(15) ρ2=2×r×α−α 

Equations (16) and (17) are adjusted with GSR and DM, considering the current vector $x_{n}^{c}$.

(16) $${x1}_{n}^{c}=x_{n}^{c}-gsr+dm$$

where, ${x1}_{n}^{c}$ represents the modified vector resulting from the adjustments made to ${x1}_{n}^{c}$. The transformation of ${x1}_{n}^{c}$ can be expressed as per Equation (17).

(17) $${x1}_{n}^{c}=x_{n}^{c}-r\times \rho 1\times \frac{2∆x\times x_{n}^{c}\;}{{(vp}_{n}^{c}-vq_{n}^{c}+\varepsilon )\;}+r\times \rho 2\times \left(x_{best}-x_{n}^{c}\right)$$

where, $vp_{n}^{c}{\;,vq}_{n}^{c}$ correspond to $v_{n}+∆x$ and $v_{n}-∆x$, respectively. The vector $v_{n}$ is the average of two vectors, $x_{n}$ and $Z_{n+1}$.

(18) $$Z_{n+1}=x_{n}-r\times (2∆x\times x_{n})\;/\;x_{worst}-x_{best}+\varepsilon )$$

Equation (19) is used to improve both local search exploitation and global search during the discovery process. By substituting the current solution vector $x_{n}^{c}$ with the new solution vector $x_{best}$, the current solution vector ${x2}_{n}^{c}$ is obtained:

(19) $${x2}_{n}^{c}=x_{best}-r\times \rho 1\times \frac{2∆x\times x_{n}^{c}\;}{{(vp}_{n}^{c}-vq_{n}^{c}+\varepsilon )\;}+r\times \rho 2\times \left(x_{r1}^{c}-x_{r2}^{c}\right)\;$$

Subsequently, a new version of the solution $x_{n}^{c+1}$, is computed as per Equation (20).

(20) $$x_{n}^{c+1}=ra\times \left(rb\times {x1}_{n}^{c}+(1-rb)\times {x2}_{n}^{c}\right)+(1-ra)\times {x3}_{n}^{c}$$

where ra and rb are random numbers within the range [0, 1], and ${x3}_{n}^{c}$ is defined as per Equation (21).

(21) $${x3}_{n}^{c}=x_{n}^{c+1}-\rho 1\times \left({x2}_{n}^{c}-{x1}_{n}^{c}\right)$$

The Local Escaping Operator (LEO) process is a technique used in optimization algorithms to overcome local optima and enhance convergence. LEO helps algorithms swiftly move away from suboptimal solutions and explore new regions of the search space. By incorporating LEO, optimization algorithms gain the ability to find better and more efficient solutions. This operator plays a crucial role in improving the performance and effectiveness of optimization algorithms, making them more robust for solving complex optimization tasks.

#### 3.3.2. Improved Gradient-Based Optimizer Algorithm

When applying population-based metaheuristic algorithms for optimization, the time-varying inertia weight method is crucial. It improves the performance of the algorithm by providing a balanced approach between local search and global search capabilities. The value of the inertia weight is established in this study using an inertia weight technique. Researchers have taken this approach frequently, and it has produced encouraging results in terms of enhancing the algorithms’ ability to fine-tune. The weight parameter w is calculated using a mathematical equation, which helps in adjusting the algorithm’s behavior throughout the optimization process, promoting better exploration and exploitation of the search space given by Equation (22).

(22) $$w=0.5+\frac{r()}{2}$$

In the equation provided, the function r() represents a random function that generates values within the range [0, 1]. The inertia weight plays a significant role in the optimization process by allowing control over early evolution’s premature convergence. In many test suites, the use of an adequate inertia weight can result in the identification of better solutions. The algorithm can more effectively explore the search space and possibly avoid becoming stuck in local optima by dynamically modifying the inertia weight during the optimization phase to find a balance between exploration and exploitation. The proposed Equation (23) updates the inertia weight.

(23) $$x_{n}^{c+1}=w\times ra\times \left(rb\times {x1}_{n}^{c}+(1-rb)\times {x2}_{n}^{c}\right)+(1-ra)\times {x3}_{n}^{c}$$

This strategy aims to increase initial exploration and enhance local search ability as optimization progresses.

#### 3.3.3. HGEO—Hybrid Gradient Equilibrium Optimization

By iteratively increasing a parameter value within a defined range, the GBO algorithm enhances its exploration of potential solutions, leading to diversified solutions. This approach improves the algorithm’s efficiency in searching for optimal solutions while maintaining a balance between exploitation and exploration. The search space surrounding the best solution ($x_{best}$) is calculated using GSR using Equation (5). By including random behavior, the GBO algorithm incorporates a randomized exploration mechanism. This randomization increases search process diversity and makes it easier to find local optima. The best answer (xbest) and a randomly chosen solution are taken into account via a random offset in Equation (19), which aids in the algorithm’s exploration of various areas of the search space. Meanwhile, the Equilibrium optimizer (EO) algorithm focuses on enhancing the exploitation phase to deliver precise solutions. The generation rate ($\vec{a}$), an essential parameter in the EO algorithm, controls the rate at which new solutions are generated. Equation (19) defines the generation rate using a first-order exponential decay process. This gradual reduction in the generation rate over time allows the EO algorithm to converge toward more accurate solutions as the optimization progresses. By hybridizing these strategies, the GBO and EO algorithms complement each other’s strengths. The GBO algorithm explores the search space effectively through parameter adjustments and random exploration, while the EO algorithm fine-tunes the search by controlling the generation rate, leading to the discovery of precise solutions. The proposed HGEO is given as per Equation (24).

(24) $$gsr=r\times \rho 1\times \frac{2∆x\times x_{n}}{\left(x_{worst}-x_{best}+\varepsilon \right)}\times \vec{a}$$

(25) $$\mathrm{Where}\;\vec{a}={\vec{a}}_{0}e^{-\vec{k}(I-I_{0})}$$

### 3.4. SA-BiLSTM

**Figure 3:**
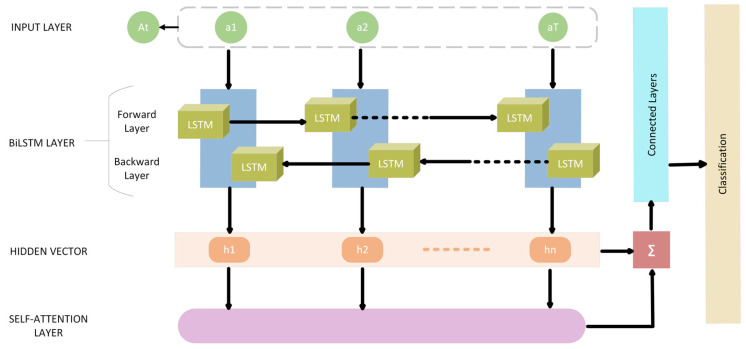
SA-Bi-LSTM.

To address the challenges posed by BiLSTM, a multi-head self-attention based deep neural network is proposed, incorporating LSTM cells. This architecture efficiently processes sequential data, identifies important features, and handles variable-length sequences. By leveraging self-attention, the model focuses on relevant parts of the input while disregarding irrelevant ones. The multi-head attention mechanism further enhances this capability by enabling parallel processing of different parts of the sequence. Figure 3 depicts the SA-BiLSTM.

LSTM’s ability to learn and remember long-term dependencies and overcome vanishing gradient problems makes it a powerful tool for sequential data processing. The proposed architecture offers advantages over BiLSTM, requiring fewer computations and providing interpretability for better understanding of predictions. A cell memory state and three gates are also present. According to Equations (26)–(31), each LSTM cell’s computation is conducted. The processes inside the LSTM cell are expressed as $A_{t}$, representing the current input vector, $h_{t-1}$ representing the most recent hidden state and $c_{t-1}$ representing the most recent memory cell state.

The input sequence $A_{t}$ undergoes processing through a Bidirectional LSTM, where it is examined both from left-to-right (Forward LSTM) and right-to-left (Backward LSTM). The outputs from these two orientations are subsequently concatenated to form a unified sequence of hidden states. Following this, a multi-head attention mechanism is employed. The unified hidden states are segmented into multiple “heads”, and attention weights are independently determined for each head. These weights are then used to compute a weighted sum of the hidden states, represented by $y_{t}$. Finally, the attention-informed hidden states pass through fully connected layers, resulting in the model’s classification output, as shown in Algorithm 2.
**Algorithm 2:** Proposed Model (SA-BiLSTM)1.InputSequence of vectors $A_{t}$ (a1, a2, …, aT)2.Bidirectional LSTM ProcessingFor_LSTM: Processing input sequence from left to right.Back_LSTM: Processing input sequence from right to left.Concatenate Outputs: Form a single sequence of hidden states.3.LSTM Cell Computation:The Input Gate is computed via Equation (26)Forget gate is calculated via Equation (27)The output gate and Input Modulation gate are calculated via Equations (28) and (29)Memory cell is updated via Equation (30)The Hidden state is updated via Equation (31)4.Multi-Head Attention MechanismHidden States are divided into multiple “heads”.Attention Weights calculated Individually for each head, and weighted sum is calculated.Weighted Sum $y_{t}=\Sigma (\alpha_{t}\;*\;h_{t})$, where Σ represents the elementwise multiplication and sum of attention weights and hidden states.5.Fully Connected LayersProcess the attention-weighted hidden states through one or more fully connected layers.6.OutputClassification, including attention-weighted hidden states.

(26) $${inp}_{t}=S_{a}(I_{A.Inp}A_{t}+R_{h.inp}h_{t-1}+b_{v}(inp)$$

(27) $${fg}_{t}=S_{a}(I_{A.Fg}A_{t}+R_{h.Fg}h_{t-1}+b_{v}(Fg)$$

(28) $${out}_{t}=S_{a}(I_{A.Out}A_{t}+R_{h.Out}h_{t-1}+b_{v}(Out)$$

(29) $$m_{t}=B(I_{At}A_{t}+R_{hc}h_{t-1}+b_{v}(c)$$

(30) $$c_{t}={Fg}_{t}c_{t-1}+m_{t}.{Inp}_{t}$$

(31) $$h_{t}={Out}_{t}.B(c_{t})$$

At time t, the symbols where ${inp}_{t}$, ${fg}_{t}$, ${out}_{t}$, and $m_{t}$ are used to denote the input, forget, output, and input modulation gates, respectively. The input weights are symbolized by $I_{A}$, the recursive weights by R, and the bias vector by $b_{v}$. The sigmoid activation function is given by $S_{a}\left(A\right)={\left(1+e^{-A}\right)}^{-1}$, and $B\left(A\right)=\left(e^{A}-e^{-A}\right)/\left(e^{A}+e^{-A}\right)$ represent the hyperbolic tangent function. The vector ${fg}_{t}$ which supplies potential values for the memory cell’s update, is derived from the current input and preceding state through the application of the tanh activation function. Forget gate ${fg}_{t}$ plays a role in discarding the data that were previously communicated. The output gate ${out}_{t}$ holds the information for subsequent operations that govern the cell’s output at time t. The hidden state $h_{t}$ is calculated using the elementwise multiplication of the output gate vector ${out}_{t}$ and the current memory cell state ${out}_{t}$, after being projected by the tanh function. Following this, the memory cell referred to as $c_{t}$, is updated.

Bidirectional LSTMs and multi-head attention are two potent deep learning approaches that are combined in the Multi-Head Attention-based Bidirectional LSTM (MHA-BiLSTM) architecture. Recurrent neural networks with the ability to process sequences both forward and backward are known as bidirectional LSTMs. This capability enables the network to collect both past and future data. Contrarily, a technique known as multi-head attention enables the network to concentrate on various elements of the input sequence at once, enhancing its capacity to model long-distance dependencies. A bidirectional LSTM layer processes the input sequence in the MHA-BiLSTM architecture first in order to capture the temporal dependencies in the data. After that, a single sequence of hidden states is created by concatenating the outputs from the forward and backward directions. The network is then given multi-head attention to the hidden states to enable it to concentrate on various sections of the sequence. This is accomplished by dividing the hidden states into many “heads” and computing attention weights for each head individually. The weighted sum of the hidden states created by combining the attention weights is then used as the input for the network’s next layer. The ultimate output of the network is generated by feeding the output of the attention layer into one or more fully connected layers. The final output of the SA-BiLSTM is obtained by combining the attention-weighted hidden states, as in Equation (32):

(32) $$y_{t}=\Sigma (\alpha_{t}\times h_{t})$$

where, Σ represents the sum of the elementwise multiplication of the attention weights $\alpha_{t}$ and the corresponding hidden states ($h_{t}$).

## 4. Result and Discussion

### 4.1. Dataset Description

The datasets that are used in the model for evaluation are the KDD-CUP99 and TON_IoT datasets. KDD-CUP99 [29] is considered a benchmark in the field of Intrusion Detection developed by DARPA. It has a wide range of intrusions mixed with regular connections. It still finds its way into modern intrusion detection applications because of the significance of the dataset. DoS is one of the main targets for this research through this dataset that will be focused on primarily. Researchers use this dataset to develop and evaluate machine learning algorithms for effective intrusion detection in computer networks. More such new datasets have come up, which not only have recent data collection but also have data collection on the Internet of Things (IoT) and the Industrial Internet of Things (IIoT). The TON_IoT dataset [30] is a new-generation dataset of such things, designed for evaluating cybersecurity applications based on AI and Machine/Deep Learning algorithms. These datasets encompass heterogeneous data from IoT and IIoT telemetry datasets, Windows and Linux operating system datasets, and network traffic datasets. Gathered from a realistic and large-scale network environment at UNSW Canberra, the datasets feature a testbed network with IoT and IIoT networks, along with simulated cyber-attacks like DoS, DDoS, and ransomware. Data sets are available for researchers to discuss and analyze cybersecurity projects. These include intrusion detection, malicious software detection, privacy protection, digital and criminal activity detection, and tracking potential security threats. The VeReMi dataset [31] is a dataset that suits a VANET architecture and simulates it perfectly for a vehicular attack scenario. The benchmark dataset is a standard dataset that consists of various attacks, but for our use, we have combined all attacks into one class. The purpose of combining all attacks is to create an environment that has two classes: the attack class and the normal class.

It is clarified that the KDD-CUP99 is an older dataset, and the nature of the network has evolved since then. Our intention behind using it was to check our algorithm’s performance on diverse datasets, including legacy data. Similarly, the TON_IoT dataset was used as a more recent dataset to see how our algorithm performs with contemporary network traffic. It has been clarified that two of the datasets are not directly representative of the VANET environment. However, our aim was to show that our method’s applicability is not limited to a specific type or era of network traffic. To emulate the VANET environment, we have used the VeReMi dataset.

### 4.2. Experiment Setup

Our experimental setup is backed by a robust Intel (R) Core (TM) i7-8700 CPU (IBM India, Delhi, India) clocked at 3.20 GHz. The system boasted 16 GB of RAM, ensuring efficient multitasking and data handling. Furthermore, the presence of a 256-GB SSD is used for faster data access. The experiments were performed on a Windows 11 operating system using MATLAB 2019a. ReLU is used as an activation function. For training the model, we adopted a learning rate of 0.001 and tested with different epochs, i.e., 100, 200, 300, 400, and 500. All calculations were batch-processed in blocks of 512 for better convergence.

### 4.3. Performance Analysis

The performance of the CCNN-SA-BiLSTM is analyzed, and its results are compared to those of internal adaptations and variations of our proposed algorithm, i.e., CCNN-BiLSTM, CCNN-LSTM, and other established algorithms, CNN-LSTM, CNN-GRU (Gated recurrent unit), and 3-LSTM. Evaluation of the suggested model’s efficacy is carried out in terms of NPV, FPR, FNR, and MCC, as well as Accuracy, Precision, Recall, Sensitivity, and Specificity. Performance is evaluated using a Confusion Matrix, which includes metrics such as ACC (Accuracy), PRE (Precision), SEN (Sensitivity), SPE (Specificity), F_M (F-measure), NPV (Negative Predictive Value), FPR (False Positive Rate), FNR (False Negative Rate), and MCC (Matthew’s correlation coefficient). With the help of individual metrics, a confusion matrix is leveraged at the end to provide a consolidated view of the model’s performance. The methodology for calculating these metrics is described in detail in this section.

1. Accuracy
  The percentage of accurately predicted cases in all examples is used to measure accuracy.
    $$ACC=\frac{Tp+Tn}{Tp+Fp+Fn+Tn}$$

2. Precision
  Precision is a helpful indicator of how precisely the positive chemicals are expected because it indicates the percentage of correctly anticipated positive cases in all test findings.
    $$PRE=\frac{Tp}{Tp+Fp}$$

3. Sensitivity
  By dividing the total positives by the percentage of genuine positive forecasts, one may determine the sensitivity number, also referred to as Recall.
    $$SEN=\frac{Tp}{Tp+Fn}$$

4. Specificity
  The percentage of successfully predicted negative outcomes over all negative outcomes is known as specificity.
    $$SPE=\frac{Tn}{Tn+Fp}$$

5. F-Measure
  In order to ensure that each class only contains a single sort of data item, the F-Measure number strikes a compromise between fully identifying all data bits and doing so.
    $$F\_M=\frac{PRE.\;SEN}{PRE+SEN}$$

6. Matthew’s correlation coefficient (MCC)
  A binary two-by-two variable association measure is the MCC, also called the Phi Coefficient.
    $$MCC=\frac{\left(Tp\times Tn-Fp\times Fn\right)}{\sqrt{(Tp+Fn)(Tn+Fp)(Tn+Fn)\left(Tp+Fp\right)}}$$

7. Negative Prediction Value (NPV)
  The performance of a diagnostic test or similar quantitative metric is described by NPV.
    $$NPV=\frac{Tn}{Tn+Fn}$$

8. False Positive Ratio (FPR)
  The false positive rate is derived by dividing the total number of negative events by the total number of negative events that were incorrectly labeled as positive (false positives).
    $$FPR=\frac{Fp}{Fp+Tn}$$

9. False Negative Ratio (FNR)
  The “false-negative rate,” sometimes known as the “miss rate,” is the probability that the test will fail to identify a real positive.
    $$FNR=\frac{Fn}{Fn+Tp}$$

The results in Table 2 show that the proposed CCNN-SA-BiLSTM model outperforms all other models on the KDD’99 dataset. It achieves a high sensitivity of 99% and a high specificity of 100%, indicating its ability to accurately detect both true positive and true negative instances. The overall accuracy of the proposed model is 99%, demonstrating its capability to correctly classify intrusion and non-intrusion instances. Furthermore, the precision of CCNN-SA-BiLSTM is 99%, indicating that the model is effective at identifying positive instances while minimizing false positives. The F-measure of 99% confirms the model’s balanced performance between precision and sensitivity. Additionally, the NPV of 99% suggests that the model accurately identifies non-intrusion instances. The proposed model also exhibits an exceptional MCC of 98%, indicating a strong correlation between predicted and true values. Moreover, the FPR and FNR values are impressively low at 0% and 1%, respectively, signifying minimal misclassifications of positive and negative cases.

Table 3 presents the performance comparison of intrusion detection models on the TON_IoT dataset. The results demonstrate that the proposed CCNN-SA-BiLSTM model maintains a high level of performance on the TON_IoT dataset. It achieves a sensitivity of 99% and a specificity of 99%, indicating its ability to accurately detect both positive and negative instances of intrusion. The overall accuracy of the model is 99%, showcasing its capability to correctly classify instances in the dataset. Moreover, the precision of CCNN-SA-BiLSTM is 99%, highlighting its effectiveness in identifying positive instances while minimizing false positives. The F-measure of 99% further corroborates the model’s balanced performance between precision and sensitivity. Additionally, the NPV of 99% indicates its proficiency in accurately identifying non-intrusion instances. Furthermore, the MCC value of 98% suggests a strong correlation between the predicted and true values, indicating the model’s reliability in making accurate predictions. The FPR and FNR values are both low at 1%, indicating a minimal rate of misclassification for both positive and negative instances. Among the existing models, CCNN-BiLSTM shows the closest performance to the proposed model, with high sensitivity, specificity, and accuracy scores. However, the proposed CCNN-SA-BiLSTM model outperforms all other models across most metrics, demonstrating its superior intrusion detection capability on the TON_IoT dataset.

Table 4 presents the performance comparison of various algorithms; the Proposed Algorithm exhibited an accuracy of 98.6%. Its precision and sensitivity stood at 97.8%, with an F-measure of 96.1% [35]. While CNN-LSTM [36] registered a high precision of 99.6%, its sensitivity was slightly lower at 95.6%, resulting in an F-measure of 97.6%. The CCNN-BiLSTM method followed closely, with metrics showing 97.2% accuracy, 96.3% precision and sensitivity, and a 93.4% F-measure. The CCNN-LSTM showed a similar performance, with 97.1% accuracy and an F-measure of 92.1%. Among the methods, 3-LSTM [36] had the lowest metrics, with 95% accuracy and an F-measure equal to its sensitivity of 94.95%.

It is evident that while the Proposed Algorithm and CNN-LSTM [36] perform closely, the former provides a more balanced performance across all metrics.

### 4.4. Graphical Representation

**Figure 4:**
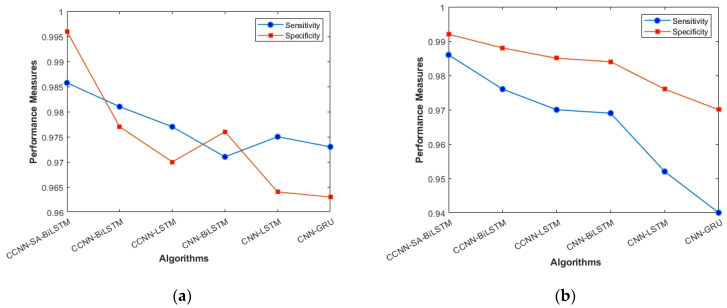
Sensitivity and Specificity (**a**) KDD’99; (**b**) TON_IoT.

**Figure 5:**
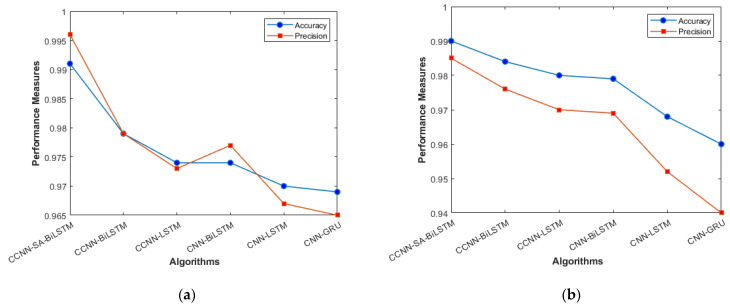
Accuracy and Precision (**a**) KDD’99; (**b**) TON_IoT.

**Figure 6:**
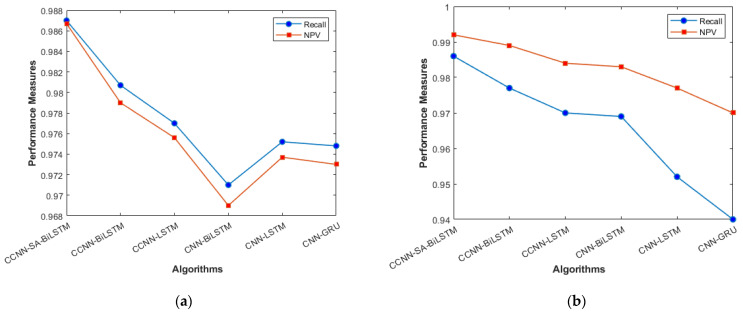
Recall and NPV (**a**) KDD’99; (**b**) TON_IoT.

**Figure 7:**
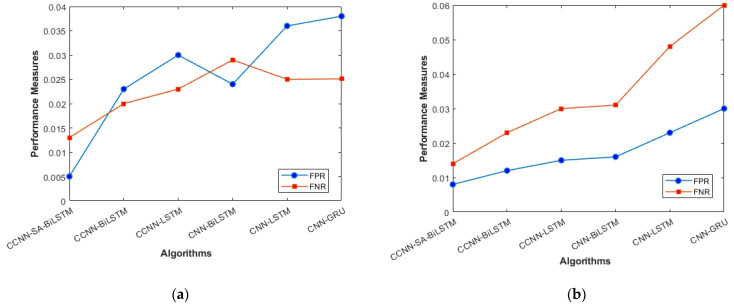
FPR and FNR (**a**) KDD’99; (**b**) TON_IoT.

**Figure 8:**
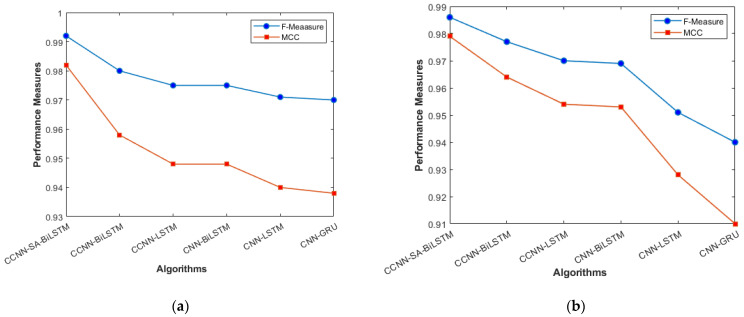
F_Measure and MCC (**a**) KDD’99; (**b**) TON_IoT.

**Figure 9:**
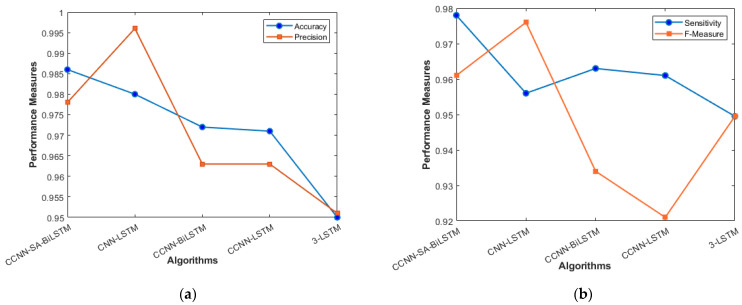
VeReMi Dataset (**a**) Accuracy and Precision; (**b**) Sensitivity and F_Measure.

**Figure 10:**
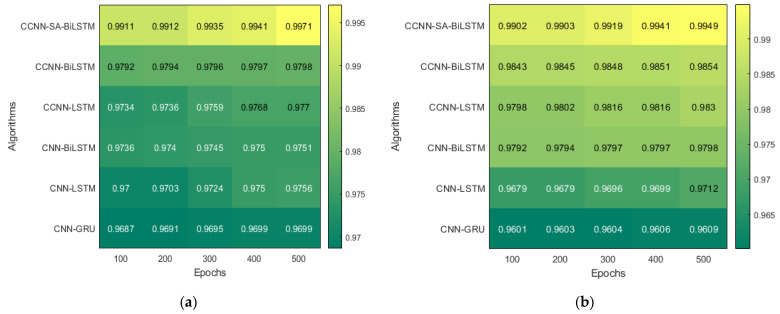
Epochs (**a**) KDD’99; (**b**) TON_IoT.

**Figure 11:**
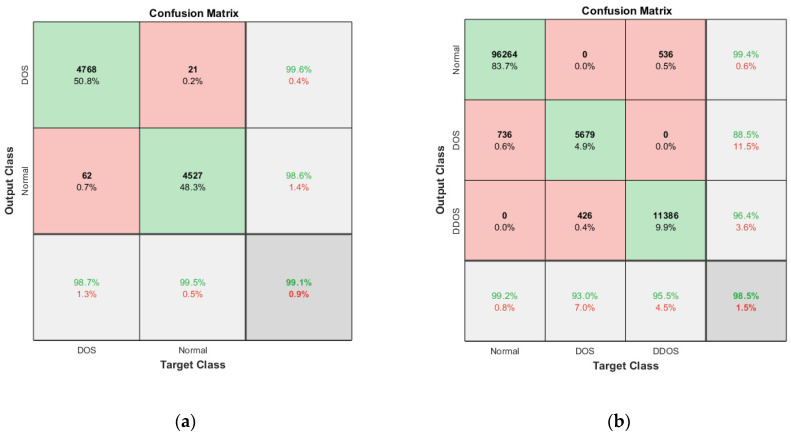
Confusion Matrix: (**a**) KDD’99; (**b**) TON_IoT.

**Figure 12:**
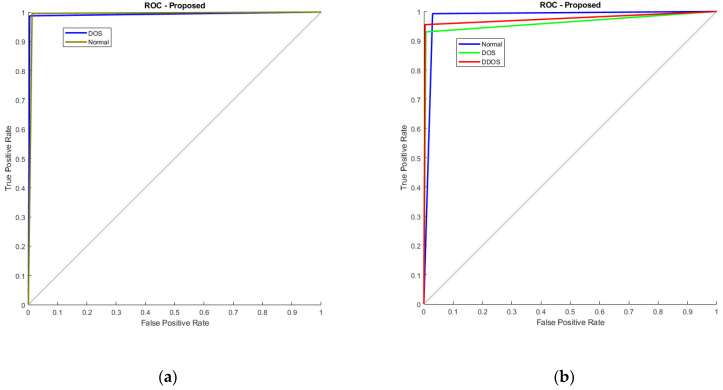
RoC Curve (**a**) KDD’99; (**b**) TON_IoT.

The performance comparison between the current models and the suggested model for KDD’99, TON_IoT, and VeReMi datasets are shown graphically in Figure 4, Figure 5, Figure 6, Figure 7, Figure 8, Figure 9, Figure 10, Figure 11 and Figure 12. The *y*-axis displays the values of the performance metrics, while the *x*-axis indicates the various models. Each statistic is represented by two subplots in the graph, one for KDD’99 and the other for the TON_IoT dataset. The graph shows that the suggested model performs better than the current models across all performance criteria for the two datasets. The suggested model’s accuracy is superior to the current models for both datasets. For both datasets, the suggested model’s FNR and FPR are lower than those of the current models. For KDD’99, the suggested model outperforms the current models in terms of MCC and NPV, but for the TON_IoT dataset, the proposed model outperforms the existing models in terms of MCC and NPV. The suggested model has better Precision, Sensitivity, and Specificity than the current models for both datasets. The VeReMi dataset is considered in Figure 9.

Figure 4a, b represents the sensitivity and specificity of the KDD’99 and TON_IoT Datasets. The balance between true positive and true negative is shown using this graph, wherein the proposed model (CCNN-SA-BiLSTM) performed better than others. Figure 5a, b represents the accuracy and precision of the different models on different datasets. It shows the overall classification of the model. The accuracy of the proposed model is the best among all the models, which is attributed to the Bidirectional nature of the model, which considers both past and future data and results in good predictions.

Figure 6a, b represents the Recall and NPV results, indicating the importance of identifying True Positives and True negatives. The high result indicates the extent to which the model was able to identify both factors. CCCN-SA-BiLSTM outperforms the other models by a margin of 1%. The identification of mistakes made by the classifier is equally important, as it helps in tuning the model’s threshold for classification. Figure 7a, b are used for this purpose, and the metrics used here are False Positive Rate and False Negative Rate.

The overall holistic view of the model is finally represented by F_Measure and MCC, which are represented in Figure 8a, b. The overall balance between precision, Recall, and correlation is shown, and the model (CCNN-SA-BiLSTM) performed better than the others. The classes are balanced, which is one of the factors that leads to low FPR and FNR for our proposed work, which can be seen in Figure 11 as well. The cascaded CNN captures the hierarchical features effectively, reducing the FPV and FNV significantly. The self-attention mechanism in our work enhances the long-term dependencies, which leads to better results when compared with other existing models.

Figure 9a represents the accuracy and precision of proposed and existing models. The accuracy of our model stands at 98.6%, which slightly outperformed the CNN-LSTM model by less than 1%. The LSTM model surely improves the accuracy of the model in the VANET architecture, as can be seen in Figure 9. The addition of the Self- attention Layer has given some edge to our model in terms of accuracy, but when compared with precision metrics, CNN-LSTM has an advantage over the proposed model. Figure 9b talks about the sensitivity and F_Measure of the models. The stacked 3-LSTM performs the lowest among all the models. The cascaded way of using LSTM does not improve the result as much as cascaded CNN, which is evident in the graph when sensitivity is taken into consideration.

A performance comparison of accuracy between the proposed and existing models is presented in Table 5 and Table 6.

The model runs for five different Epoch values, and the result pertaining to that is mentioned above. The result with a 500-epoch value showed promising results in both datasets. These tables illustrate the variations in performance (accuracy) across different epochs for the KDD’99 and TON_IoT datasets, respectively. The accuracy improved as the epochs were increased, which helped the model converge at a better local minimum value. Further, an increase in epoch would have resulted in overfitting of the dataset.

The heatmap shown in Figure 10, extracted from Table 5 and Table 6, shows the accuracy with five different epochs, where (a) represents KDD’99 and (b) represents the TON_IoT dataset. The accuracy of the DoS class in the KDD’99 dataset as per the confusion matrix is 99.6%, whereas that of the normal class is 98.6%, as represented in Figure 10a.

Likewise, for the TON_IoT Dataset, the accuracy of the Normal class is 99.4%, and that of the DoS and DDoS classes is 88.5% and 96.4%, as represented in Figure 11b. The low values of False Positive and False negative help achieve good performance in the model. The class imbalance factor is also taken into consideration, as can be seen in the table, where a roughly equal distribution of all the classes is present in the model. Thus, the proposed model exhibits robust performance on the given datasets.

Additionally, Figure 12 illustrates the RoC curve, with (a) denoting KDD’99 and (b) representing TON_IoT. The RoC plots the true positive rate against the false positive rate at various thresholds. In KDD’99, the AUC value is nearing 1, representing the classifier is good at differentiating among both the classes, whereas in TON_IoT, one vs. all analysis is used, wherein the first normal class is taken as positive and the rest as negative, the next being DoS vs. all, and finally DDoS vs. all. The AUC of 0.98, 0.975, and 0.965 represent that the model is able to clearly distinguish between the classes and represents good model performance.

The performance of the CCNN-SA-BiLSTM is attributed to the integration of the SA mechanism with the BiLSTM. The self-attention mechanism enables the model to focus on the most relevant features, while the bidirectional nature of the LSTM captures patterns from both past and future time steps. This combination allows for a more comprehensive feature understanding, leading to better classification performance as compared to other methods. The result is supported by the different metrics discussed above, and the results for the same are depicted via graphs.

## 5. Conclusions and Future Work

Vehicle problems directly affect both human and traffic safety, making the security of the vehicle network a significant and important issue. Recent years have seen the importance of VANET grow for enabling intelligent transport systems, ensuring traffic safety, and avoiding collisions. VANETs, however, encountered serious difficulties as a result of several attacks, such as DoS and DDoS. A strong AI-based NIDS was required to overcome these security issues. This research presents an innovative method for creating an AI-based NIDS that makes use of deep learning techniques. The proposed model specifically included SA-BiLSTM for classification and CCNN for learning high-level features. CCNN and SA-BiLSTM performance was improved using the Multi-variant of Gradient optimization algorithm (MV-GBO). Additionally, feature learning was improved by using information gained using MV-GBO-based feature extraction. On the MATLAB platform, trustworthy datasets like KDD-CUP99, ToN-IoT, and VeReMi were used to assess the effectiveness of the suggested model. In future work, the model will be tested on more VANET network beds to check the robustness of the model and its adaptability, as well as on more attacks and faults. Integrating the model with edge computing to reduce the latency in decision-making and processing will further enhance the work. The model’s scalability to handle large network traffic without compromising the model’s accuracy will also be explored in the future by the team.

## Figures and Tables

**Table 1:** Abbreviations.

Symbol	Abbreviations
${Cl}_{i}$	Class *i*
$H\left(B\right)$	Entropy of random variable *B*
$P({Cl}_{i})$	Probability of Class *i*
$I_{j}$	Feature (*j* is an index)
$H\left(B|I_{j}\right)$	Entropy of random variable *B*, given $I_{j}$
$x_{n}$	*n^th^* initial vector of D dimensional space
$x_{min}$	Decision variable lower bound
$x_{max}$	Decision variable upper bound
gsr	Global search radius
rn	Random Number
ε	Constant to ensure numerical stability
α	Control parameter
β	Probability rate to balance GBO Algorithm
r	Random number
ra	Random number within [0, 1]
rb	Random number within [0, 1]
ρ2	Parameter to modify phase size of vector
$v_{n}$	Average of two vectors
dm	Directional movement to converge
$\vec{a}$	Generation rate
$x_{n}^{c}$	A solution space for current iteration *c*

**Table 2:** Proposed and existing models’ performance comparison: KDD’99.

Methods	SEN	SPE	ACC	PRE	F_M	NPV	FPR	FNR	MCC
Proposed CCNN-SA-BiLSTM	0.99	1.00	0.99	1.00	0.99	0.99	0.00	0.01	0.98
CCNN-BiLSTM	0.98	0.98	0.98	0.98	0.98	0.98	0.02	0.02	0.96
CCNN-LSTM	0.98	0.97	0.97	0.97	0.97	0.98	0.03	0.02	0.95
CNN-BiLSTM	0.97	0.98	0.97	0.98	0.97	0.97	0.02	0.03	0.95
CNN-LSTM [32]	0.98	0.96	0.97	0.97	0.97	0.97	0.04	0.02	0.94
CNN-GRU [33]	0.97	0.96	0.97	0.96	0.97	0.97	0.04	0.03	0.94

**Table 3:** Proposed and existing models’ performance comparison: TON_IoT.

Methods	SEN	SPE	ACC	PRE	F_M	NPV	FPR	FNR	MCC
Proposed CCNN-SA-BiLSTM	0.99	0.99	0.99	0.99	0.99	0.99	0.01	0.01	0.98
CCNN-BiLSTM	0.98	0.99	0.98	0.98	0.98	0.99	0.01	0.02	0.96
CCNN-LSTM	0.97	0.98	0.98	0.97	0.97	0.98	0.02	0.03	0.95
CNN-BiLSTM	0.97	0.98	0.98	0.97	0.97	0.98	0.02	0.03	0.95
CNN-LSTM [34]	0.95	0.98	0.97	0.95	0.95	0.98	0.02	0.05	0.93
CNN-GRU [33]	0.94	0.97	0.96	0.94	0.94	0.97	0.03	0.06	0.91

**Table 4:** Proposed and existing models’ performance comparison: VeReMi.

Methods	ACC	PRE	SEN	F_M
Proposed CCNN-SA-BiLSTM	0.986	0.978	0.978	0.961
CNN-LSTM [36]	0.98	0.996	0.956	0.976
CCNN-BiLSTM	0.972	0.963	0.963	0.934
CCNN-LSTM	0.971	0.963	0.961	0.921
3-LSTM [37]	0.95	0.951	0.9495	0.9495

**Table 5:** Proposed and existing models’ performance comparison by varying Epochs: KDD’99.

Number of Epochs	100	200	300	400	500
Proposed CCNN-SA-BiLSTM	0.991149	0.991218	0.993498	0.994139	0.997091
CCNN-BiLSTM	0.979207	0.979353	0.979585	0.979748	0.979765
CCNN-LSTM	0.973448	0.973573	0.975947	0.976786	0.977019
CNN-BiLSTM	0.973555	0.974021	0.974493	0.974956	0.975111
CNN-LSTM	0.970036	0.970311	0.972446	0.975023	0.975630
CNN-GRU	0.968650	0.969053	0.969457	0.969892	0.969947

**Table 6:** Proposed and existing models’ performance comparison by varying Epochs: TON_IoT.

Number of Epochs	100	200	300	400	500
Proposed CCNN-SA-BiLSTM	0.990159	0.990293	0.991935	0.994126	0.994933
CCNN-BiLSTM	0.984323	0.98446	0.984845	0.985076	0.985405
CCNN-LSTM	0.979761	0.980202	0.981626	0.981631	0.983011
CNN-BiLSTM	0.979234	0.979386	0.979723	0.979737	0.979812
CNN-LSTM	0.967857	0.967945	0.969606	0.969872	0.971189
CNN-GRU	0.960096	0.96026	0.960357	0.960643	0.96086

## Data Availability

Research data will be available on individual requests to the corresponding author considering collaboration possibilities with the researcher or research team and with restrictions that the data will be used only for further research in the related literature progress.
